# Genetic Background Effects on the Expression of an Odorant Receptor Gene

**DOI:** 10.3389/fncel.2021.646413

**Published:** 2021-02-25

**Authors:** Artur Guazzelli Leme Silva, Maira Harume Nagai, Thiago Seike Nakahara, Bettina Malnic

**Affiliations:** Department of Biochemistry, University of São Paulo, São Paulo, Brazil

**Keywords:** DNA methylation, odorant receptor gene, olfactory receptor gene, genetic background, gene expression, single nucleotide polymorphism

## Abstract

There are more than 1000 odorant receptor (OR) genes in the mouse genome. Each olfactory sensory neuron expresses only one of these genes, in a monoallelic fashion. The transcript abundance of homologous OR genes vary between distinct mouse strains. Here we analyzed the expression of the OR gene *Olfr17* (also named P2) in different genomic contexts. *Olfr17* is expressed at higher levels in the olfactory epithelium from 129 mice than from C57BL/6 (B6) mice. However, we found that in P2-IRES-tauGFP knock-in mice, the transcript levels of the 129 *Olfr17* allele are highly reduced when compared to the B6 *Olfr17* allele. To address the mechanisms involved in this variation we compared the 5′ region sequence and DNA methylation patterns of the B6 and 129 *Olfr17* alleles. Our results show that genetic variations in *cis* regulatory regions can lead to differential DNA methylation frequencies in these OR gene alleles. They also show that expression of the *Olfr17* alleles is largely affected by the genetic background, and suggest that in knock-in mice, expression can be affected by epigenetic modifications in the region of the targeted locus.

## Introduction

Odorants are detected by a large family of odorant receptors (ORs) expressed in the olfactory sensory neurons of the nose (Buck and Axel, 1991). In the mouse genome, there are more than 1000 different OR genes, which are spread over almost all chromosomes (Zhang and Firestein, 2002; Godfrey et al., 2004). A recent comprehensive analysis found that there are in total 1141 protein-coding OR genes plus 342 OR pseudogenes in the mouse genome (Barnes et al., 2020). Each olfactory sensory neuron in the olfactory epithelium expresses one single OR gene out of the complete repertoire, through a tight mechanism of gene regulation, which is not completely understood (Chess et al., 1994; Malnic et al., 1999; Monahan and Lomvardas, 2015; Nagai et al., 2016).

Different members of the OR gene family show unequal levels of gene expression in the olfactory epithelium (Young et al., 2003; Zhang et al., 2004; Rodriguez-Gil et al., 2010; Khan et al., 2011; Ibarra-Soria et al., 2017). In addition, transcript abundance of homologous OR genes vary extensively between different mouse strains (Ibarra-Soria et al., 2017). Little is known about the mechanisms involved in this variation.

Odorant receptor gene expression encompasses various levels of regulation, involving both regulatory DNA sequences and epigenetic modifications (Monahan and Lomvardas, 2015; Nagai et al., 2016; Degl’innocenti and D’errico, 2017). *Cis* acting regulatory elements like the H and P enhancers were shown to play important roles in OR gene expression (Serizawa et al., 2003; Bozza et al., 2009; Khan et al., 2011). OR gene promoter regions and enhancer sequences are enriched in binding motifs for homeodomain and O/E-like transcription factors (Vassalli et al., 2002; Rothman et al., 2005; Hoppe et al., 2006; Michaloski et al., 2006; Hirota et al., 2007; Nishizumi et al., 2007; Young et al., 2011; Cichy et al., 2019) and motifs for other types of transcription factors (Clowney et al., 2011; Michaloski et al., 2011; Markenscoff-Papadimitriou et al., 2014; Iwata et al., 2017). These motifs are thought to influence OR gene expression frequency in olfactory sensory neurons (Michaloski et al., 2006; Khan et al., 2011; Vassalli et al., 2011; Plessy et al., 2012; Zhang et al., 2016).

Genetic variations in *cis* regulatory regions were shown to affect OR gene expression in different mouse strains (Ibarra-Soria et al., 2017). Sequence variants generated by single nucleotide polymorphisms (SNPs) may influence transcription factor-binding affinities. Also, binding of transcription factors to their respective DNA binding motifs can be affected, either, negatively or positively, by cytosine methylation (Yin et al., 2017). Even though there is evidence that DNA methylation plays a role in global gene expression in the olfactory epithelium (Macdonald et al., 2005, 2010; Colquitt et al., 2013, 2014), the involvement of DNA methylation in OR gene regulation has been poorly investigated so far.

Here we show that the OR gene *Olfr17* (also named P2) is expressed at different levels depending on the genomic context. While *Olfr17* is expressed at higher levels in the olfactory epithelium from 129 mice than from B6 mice, in P2-IRES-tauGFP knock-in (P2-GFP) mice, the transcript levels of the 129 *Olfr17* allele are highly reduced when compared to the B6 *Olfr17* allele. We found that the *Olfr17* allele from the 129-mouse strain contains SNPs leading to the occurrence of two additional CpG dinucleotides in the 5′ region. As a result, the 5′ region of the 129 *Olfr17* allele shows higher levels of DNA methylation than the one from the B6 *Olfr17* allele. The levels of DNA methylation in the 129 *Olfr17* allele are not reduced in neurons expressing 129 *Olfr17*. Finally, we found that the transcript levels of other OR genes that are in *cis* and close to the targeted locus, are also reduced in the P2-GFP mice.

## Materials and Methods

### Animal Procedures

The mouse strains used were 129S1/SvlmJ, C57BL/6 and the knock-in strain *Olfr17*tm7Mom/MomJ (P2-IRES-tauGFP) (Stock number: 006669, obtained from The Jackson Laboratory). As previously described (Feinstein and Mombaerts, 2004), the P2-GFP mice express the *Olfr17* gene from the 129SvJ mouse strain containing an IRES-tauGFP 3 bp after the stop codon of the gene, and were originally generated in a 129P2/OlaHsd background and bred with a mouse carrying an Ella-cre transgene, a transgene generated in FVB/N but having been backcrossed at least four generations to C57BL/6 before this cross to excise the loxP-flanked neomycin resistance sequence and having unknown pedigree prior to that. The resulting mice, lacking the neomycin resistance cassette, were intercrossed and homozygotes were selected that were devoid of the Ella-cre transgene, and homozygous males were sent to The Jackson Laboratory for cryopreservation of sperm. Upon our request, The Jackson Laboratory performed cryo-recovery of these mice using C57BL/6J (Stock# 000664) females as the oocyte donors. We obtained heterozygous mice from The Jackson Laboratory and intercrossed them to produce the P2-GFP^+/+^, P2-GFP^+/–^, and P2-GFP^–/–^ mice analyzed in this study.

### RLM-RACE

RNA ligase-mediated rapid amplification of cDNA ends (RLM-RACE) was performed using the GeneRacer Kit^TM^ as previously described (Michaloski et al., 2006). Total olfactory mucosa RNA was purified from C57BL/6 using TRIzol (Invitrogen, Cat. No. 15596026), following the user guide workflow. The following primers were used to amplify the *Olfr17* gene 5′-UTR: P2_R: TCCTGGAGTATCAGAGTACTC and P2_R_NESTED: CAGAGCAAGAGTCAGCTGTAG.

### DNA Bisulfite Sequencing

Olfactory mucosa and liver were dissected from C57BL/6 or *Olfr17*tm7Mom/MomJ knock-in mice in PBS pH 7.4 and the genomic DNA was immediately purified using DNeasy Blood and Tissue Kit (Qiagen, Cat. No. 69504). DNA bisulfite conversion was performed using EpiTect Bisulfite Kits (Qiagen, Cat. No. 59104). The regions of interest were amplified by PCR using the bisulfite converted DNA and the following primers: *Olfr17* promoter region (Forward TGTGTTATGATTGGTATTTTTG, Reverse CCATCCCATTATCTAATAAACTC); *Olfr17* CDS 1 (Forward AGATGTTGAGTATTTTGATATT, Reverse ACATAAAAACCCAAAATCAAC); *Olfr17* CDS 2 (Forward ATAGTTTTAGTTTGTGTTGATA, Reverse CAATATTTCCTTCAACATCCT).

The amplicons were synthetized by Platinum^®^ Taq DNA Polymerase (Invitrogen) and were subcloned using the Dual promoter TA cloning^®^ kit (Invitrogen, 45-0007LT). Individual clones were sequenced using BigDye^®^ Terminator v3.1 Cycle Sequencing Kit (Life Technologies^TM^, Cat. No. 4337455) on an ABI PRISM 3100 Genetic Analyzer (Hitachi). The number of sequenced clones varied from 10 to 17 clones for the experiments with whole olfactory mucosa and from 12 to 25 clones per replica in the case of the dissociated cells (FACS experiments).

### FACS

Fluorescence-activated cell sorting (FACS) was performed as described (Leme Silva et al., 2018). Briefly, whole olfactory mucosa was dissected in liquid culture medium CMRL-1066 supplemented with EGTA (2.0 mM) and kept on ice and cell dissociation was carried out as follows. Dissected tissue was incubated with 5 μL DNase (1 U/μL, Invitrogen, Cat. No. 18068-015) for 5 min at room temperature. During incubation, the tissue was pipetted up and down using a glass Pasteur pipette with polished tip, to obtain better dissociation. After DNase treatment, the tissue was incubated with 200 μL papain (100 U/mL, Sigma No. P 4762) for each 1.0 mL of dissociation solution, for 10 min at 37°C. To remove papain, cells were centrifuged at 300 × *g* for 5 min at 4°C, and the supernatant was discarded. Centrifugation was repeated one more time, the pellet was resuspended in CMRL-1066 containing EGTA (2 mM), and pipetted up and down using a glass Pasteur pipette with polished tip to release the cells. The cellular suspension was filtered in 40 μm mesh twice, and cells were sorted with a FACS Aria III in the Core Facility for Scientific Research – University of São Paulo (CEFAP-USP/FLUIR).

### RT-qPCR

Olfactory epithelia were dissected from 6 to 8-week-old mice and total RNA was prepared using TRIzol reagent (Thermo Fisher Scientific). RT-qPCR was performed as described in Michaloski et al. (2011), using the Fast SYBR^TM^ Green Master Mix (Thermo Fisher Scientific, Cat. No. 4385612) and a 7500 Fast Real-Time PCR System (Applied Biosystems). Primer sequences for *Olfr17* were: CTCTGATACTCCAGGACAAAACC (forward) and GGATCACAGATCGCCATGTAG (reverse); for *Olfr6* were: CTTTATGTCCCTTGCCTGTACTG (forward) and ACTGGATAGCGAAGAGGCCAA (reverse); for *Olfr15* were: TGCCTTTACTACCAGTTCAGTCC (forward) and ACACCCACCATAGCTGATTGT (reverse); for *Olfr566* were: CTGTCCTCAGTATCGCCTCCT (forward) and GGGTGCTGACCGACCATATC (reverse); for *Olfr1507* were: CAAATCCGAAAGTACAGATGGCT (forward) and CGGTGGTCGTGTATGATTGTTAT (reverse); for β-actin: AAGGCCAACCGTGAAAAGATG (forward) and GTGGTACGACCAGAGGCATACA (reverse).

### *In situ* Hybridization

**FIGURE 1:**
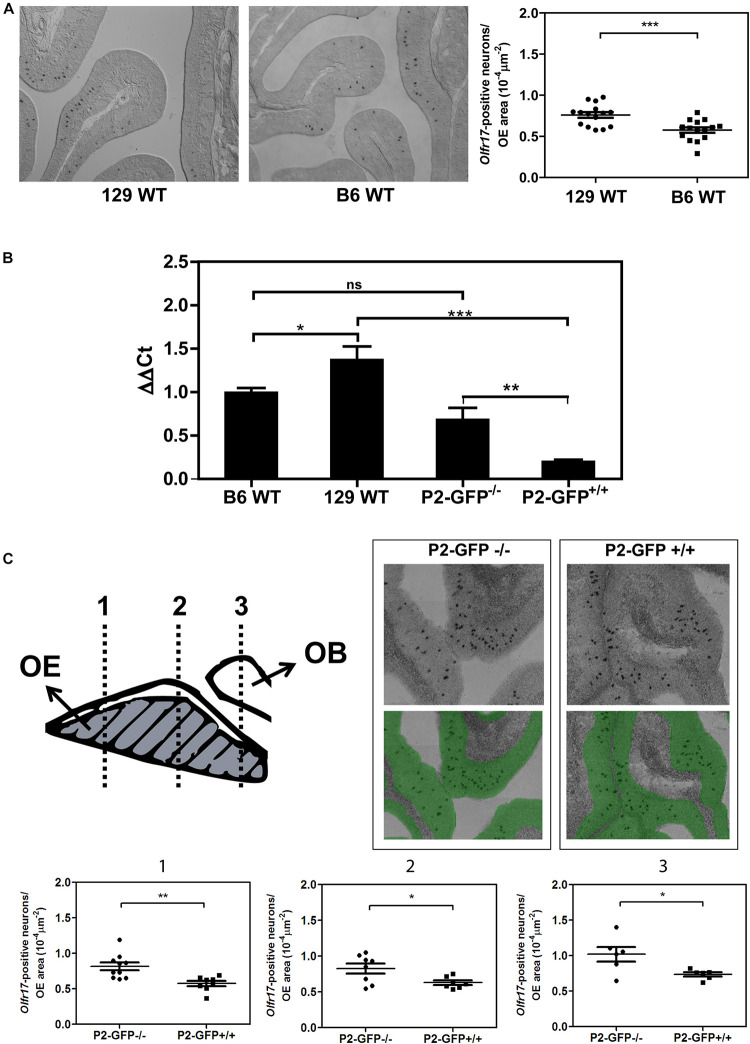
Expression of the *Olfr17* alleles in different genetic backgrounds. **(A)** Number of olfactory sensory neurons expressing *Olfr17* in the wild type mouse strains. Coronal sections through the anterior-posterior axis of the olfactory epithelium from 129 and B6 mouse strains were hybridized with antisense digoxigenin-labeled probe specific for *Olfr17*. Representative images of the labeled sections are shown. The graph shows the number of *Olfr17*-positive neurons per olfactory epithelium area. *N* = 3 animals from each strain, five sections per animal. Student’s *t*-test ***P < 0.001. **(B)** RT-qPCR was performed to determine the relative *Olfr17* gene expression levels in the olfactory epithelium from 129 and B6 wild type mouse strains, or from P2-GFP^− /−^ and P2-GFP^+/+^ knock-in siblings. The levels were normalized to β-actin and are shown relative to the expression in the wild type B6 olfactory epithelium. The graph shows mean +/− SEM (B6, *n* = 3; 129, *n* = 4; P2-GFP^− /−^, *n* = 4; P2-GFP^+/+^, *n* = 4). Not significant: ns, *P < 0.05; **P < 0.01 and ***P < 0.001. One-way ANOVA followed by Newman–Keuls *post hoc* test. **(C)** Coronal sections through the anterior-posterior axis of the olfactory epithelium from P2-GFP^− /−^ and P2-GFP^+/+^ mice were hybridized with antisense digoxigenin-labeled probe specific for *Olfr17*. Olfactory neurons that hybridized with the *Olfr17* probe were counted in 7–10 adjacent sections from each genotype from the three indicated locations using Tissue FAXS. Representative images of the labeled sections are shown on the top right. The number of labeled neurons (black dots) per olfactory epithelium area (labeled in green), was determined in each section. Student’s *t*-test, *P < 0.05 and **P < 0.01. Dots and squares represent the number of *Olfr17*-positive neurons per olfactory epithelium area in each section.

*In situ* hybridization was performed as previously described (Von Dannecker et al., 2005; Camargo et al., 2019). Sections cut through the noses of 3-week-old mice were hybridized with digoxigenin-labeled cRNA probes corresponding to the nucleotides 169–931 within the coding region of the *Olfr17* gene. For Figure 1A, the olfactory epithelium from three C57BL6/J and three 129S animals were cut in 16 μm sections parallel to the cribriform plate, in a posterior-anterior direction. Sections were collected in a serial fashion, discarding six 40 μm sections between the series. For each nose, eight different series were collected on 10 microscope slides, resulting in each slide representing all regions of the olfactory epithelium. Each section was imaged using a 10X objective lens and composite images were obtained using Image J Stitching plugin (Preibisch et al., 2009). The olfactory epithelium area for one half of each section was manually defined using Image J’s Polygon tool and the total area was calculated in μm^2^. The *Olfr17*-positive neurons were manually counted with Image J’s Multi-point tool. For Figure 1C, the olfactory epithelium was cut as above and adjacent tissue sections were collected on microscope slides, corresponding to locations 1, 2, and 3. Images of the entire *in situ* hybridization sections in Figure 1C were acquired by using the TissueFAXS System and the analysis was carried out using TissueFAXS^TM^ Cytometry. Image analysis was done by StrataQuest TissueGnostics, Austria. The number of black spots (positive *in situ* hybridization signals) per olfactory epithelium area (μm^2^, shown in green) per slice was determined.

### Statistical Analysis

Analysis was performed using the GraphPad Prism software. For more than two groups we employed one-way analysis of variance (ANOVA) followed by a Newman–Keuls *post hoc* test.

## Results

### Gene Expression Levels of *Olfr17* in Different Mouse Strains

We first determined the transcript levels of *Olfr17* in the olfactory epithelium from the wild type 129 and B6 mouse strains. *In situ* hybridization experiments showed that the number of *Olfr17* expressing olfactory neurons is higher in the 129-mouse strain (7.6 × 10^–5^ neurons/μm^2^ in 129 versus 5.8 × 10^–5^ neurons/μm^2^ in B6; Figure 1A). In addition, RT-qPCR experiments, where a pair of primers matching regions that are common to both the 129 and B6 *Olfr17* alleles were used, showed that *Olfr17* transcript levels were higher in the 129 strain than in the B6 strain (Figure 1B). These results are in agreement with previous RNA-seq experiments, where the transcriptional profiles of the complete OR repertoire was quantified in different mouse strains (Ibarra-Soria et al., 2017).

We next compared the transcript levels of *Olfr17* in the olfactory epithelium from P2-GFP mice. In these mice, the targeted *Olfr17* is derived from the 129-mouse strain, while the untargeted *Olfr17* is from the B6 strain (Mombaerts et al., 1996; Feinstein and Mombaerts, 2004). We found a significant difference in the transcript levels of the *Olfr17* alleles when the knock-in mice are compared to the wild type mouse strains. In the olfactory epithelium from P2-GFP^+/+^ mice, *Olfr17* is expressed only from 129 alleles, and the *Olfr17* transcript levels are significantly reduced when compared to the levels of the olfactory epithelium from P2-GFP^–/–^ mice, where the *Olfr17* is expressed from the B6 alleles (Figure 1B). Accordingly, we found that P2-GFP^+/+^ mice show 30% reduction in the number of *Olfr17* expressing neurons in comparison to the P2-GFP^–/–^ mice (9.1 × 10^–5^ neurons/μm^2^ and 6.3 × 10^–5^ neurons/μm^2^, respectively) (Figure 1C). These results suggest that the difference observed by RT-qPCR is due to a decreased number of neurons that express *Olfr17*, which is consistent with previous observations (Walters et al., 1995; Khan et al., 2011; Ibarra-Soria et al., 2017). It is important to note, however, that in this case, while there is a 30% decrease in the number of *Olfr17* expressing neurons in the P2-GFP^+/+^ mice (Figure 1C), there is a 64% reduction in *Olfr17* mRNA, when compared to P2-GFP^–/–^ mice (Figure 1B). These results indicate that the reduced number of *Olfr17* expressing neurons alone does not account for the reduced levels of *Olfr17* mRNA, and raises the possibility that in the knock-in animals, the levels of *Olfr17* mRNA per neuron are lower as well.

Altogether these results show that the 129 *Olfr17* allele is expressed at reduced levels in the P2-GFP mice (of mixed B6/129 genetic background) when compared to the wild type 129 mouse strain. In contrast, the B6 *Olfr17* allele is expressed at similar levels in both wild type B6 mice and knock-in mice.

### Genetic Variation in the 5′ Region of the 129 and B6 *Olfr17* Alleles

**FIGURE 2:**
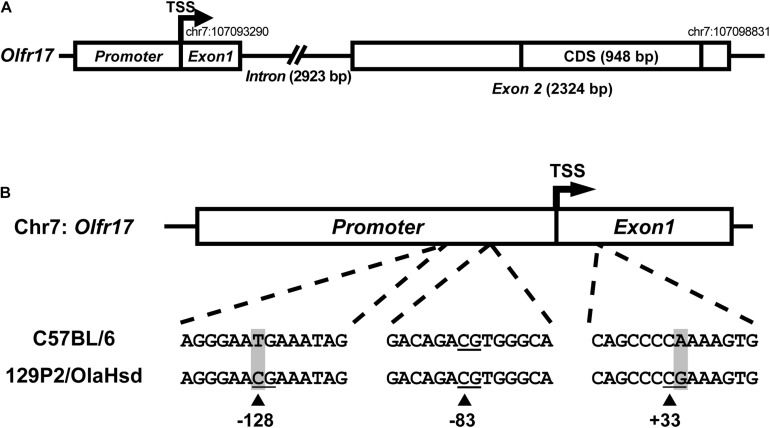
Sequence variation in the 5′ regions of the 129 and B6 *Olfr17* alleles. **(A)** Schematic representation of the *Olfr17* gene structure. The olfactory receptor gene *Olfr17* contains two exons, and the second exon contains the complete coding sequence (CDS) of the gene. The genomic coordinates are according to the GRCm38/mm10 assembly and display the positions of the transcription start site (TSS) and of the last nucleotide within the 5′-UTR. The presumed promoter region is indicated. **(B)** Schematic representation of the 5′ region of the *Olfr17* gene of the B6 and 129 mouse strain alleles showing the positions of the CpG dinucleotides. There are two single nucleotide polymorphisms (SNPs) in this region which lead to the generation of two additional CpGs in the 129 *Olfr17* allele (at positions –128 and +33).

Sequence variation in *cis* regulatory regions of the *Olfr17* alleles could account for the reduced expression levels of the 129 allele when compared to the B6 allele in the P2-GFP mice. We next determined the 5′ gene structure (exon and intron distribution) and transcription start site (TSS) of *Olfr17* by using RLM-RACE (Michaloski et al., 2006, 2011). The *Olfr17* gene shows a structure that is similar to a large number of OR genes: it is composed by two exons, which are separated by a long intron (in this case, 2923 bps long) (Figure 2A; Michaloski et al., 2006). The coding sequence (CDS, 948 bp long) is located within the second exon. The genomic position of the transcription start site (TSS) (chr7:107093290) is highly coincident with the one previously identified by using a microarray based high-throughput method (chr7:107093296) (Clowney et al., 2011).

Promoter sequences are typically located immediately upstream of the TSS. A previous large-scale study has shown that 88.5% of the OR gene promoters have a single well defined TSS position (Plessy et al., 2012). Different studies also showed that genomic sequences in the region −300 (upstream) of these OR gene TSSs are enriched for well-established OR *cis-*regulatory elements, such as the O/E and HD like sites (Michaloski et al., 2006, 2011; Clowney et al., 2011; Plessy et al., 2012). However, a very small number of minimal OR promoter sequences have been empirically validated to date, by showing that these short sequences (∼300 bp) located upstream the TSS are capable of driving punctate gene expression in the olfactory epithelium (Vassalli et al., 2002; Rothman et al., 2005; Zhang et al., 2007; Plessy et al., 2012). Based on these findings, we determined the presumed promoter region of the *Olfr17* gene (Figure 2A). Whether this sequence is able to control *Olfr17* gene transcription, still remains to be empirically determined.

By comparing the 5′ region sequences of the *Olfr17* gene derived from the 129 and B6 mouse strains, we identified the presence of two SNPs that lead to the occurrence of two additional CpG dinucleotides in the 129 *Olfr17* allele, one in the promoter region and the other one in exon 1, which are absent from the B6 *Olfr17* allele (Figure 2B). Since DNA methylation is usually involved in regulation of gene expression and typically occurs at the carbon 5 position of cytosine (5 mC) in CpG dinucleotides, we decided to investigate the methylation pattern of the *Olfr17* gene.

### DNA Methylation in *Olfr17*

We used DNA bisulfite sequencing (DNA bis-seq) to determine the DNA methylation profile in *Olfr17*. This method allows us to identify the presence of 5 mC at single-base resolution. Since cytosine methylation may vary among cells, we sequenced a large number of clones (see “Materials and Methods”) to analyze the distribution of the methylated cytosines in single molecules. To check the efficiency of the bisulfite treatment we first analyzed the methylation frequency in six CpG dinucleotides within the Gap43 promoter, which were previously shown to be highly methylated in the mature olfactory epithelium (Macdonald et al., 2010). We obtained results that are highly similar to the previous ones (Supplementary Figure 1).

**FIGURE 3:**
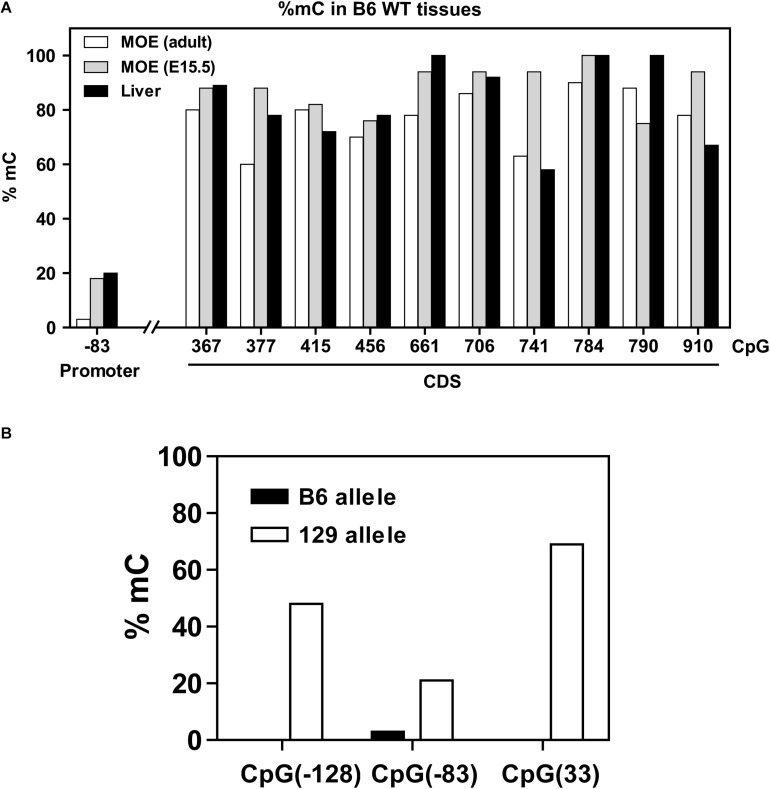
DNA methylation in the *Olfr17* gene. **(A)** The CpG methylation frequencies in the promoter and coding regions of the *Olfr17* gene are shown. The frequencies are given by the number of clones that retain cytosine after bisulfite conversion in each position divided by the total number of clones analyzed. *Olfr17* cytosine methylation was analyzed in olfactory mucosa from adult mice (MOE, adult), olfactory mucosa from E15.5 mouse embryos (MOE, E15.5) and liver. **(B)** Comparison of the methylation frequencies of the CpGs in the *Olfr17* promoter region from the B6 and 129 alleles in the P2-GFP mice.

Methylation results obtained for the 5′ region and coding region of the *Olfr17* gene in the olfactory mucosa from B6 mice are shown in Figure 3A. CpGs within the coding region of the gene show high frequencies of cytosine methylation (varying from 65 to 94%). There is only one CpG in the promoter region of the *Olfr17* gene, and this CpG shows an extremely low frequency of methylation (methylation was observed in only 6% of the analyzed sequences). We next analyzed CpG methylation in the promoter regions of the two *Olfr17* alleles in the P2-GFP mice and found that the level of CpG −83 methylation in the 129 *Olfr17* promoter region in the olfactory mucosa is higher when compared to the B6 *Olfr17* promoter region (Figure 3B). Also, the presence of the two additional CpGs contribute to an overall higher DNA methylation level in the 129 *Olfr17* promoter region when compared to the B6 *Olfr17* promoter region (Figure 3B).

We also determined the *Olfr17* gene cytosine methylation frequencies in the olfactory mucosa from E15.5 mouse embryos (which is composed mainly of immature OSNs) and in liver (where ORs are not expressed). These tissues showed similar distributions of CpG methylation, with higher frequencies in the coding region (≥58%) than in the promoter region (6 to 20%) (Figure 3A).

As mentioned above, cytosine methylation occurs in general at CpG dinucleotides, however, non-CpG methylation has been increasingly reported, although its function is not understood (Ramsahoye et al., 2000; Guo et al., 2014; Patil et al., 2014; Kinde et al., 2015). Cytosine methylation in the CpA context was previously shown to occur in an OR gene enhancer (the H region) in the olfactory mucosa (Lomvardas et al., 2006). We therefore also analyzed the distribution of CpA methylation in the *Olfr17* gene, and found that it occurs at low frequencies, both in the promoter region and coding region of the *Olfr17* gene (varying from 6 to 24%) (Supplementary Figure 2). Also, CpA methylation is randomly distributed throughout the sequence, that is, there are no specific CpAs which are preferentially methylated in the *Olfr17* gene.

### CpG Methylation of the *Olfr17* Gene From P2-GFP^+^ Neurons

CpG methylation is usually correlated with repression of gene transcription. We next asked whether the methylation pattern observed in the 5′ region of 129 *Olfr17* is reduced when the gene is expressed. The *Olfr17* DNA methylation patterns shown in Figure 3 were obtained by using genomic DNA extracted from the whole olfactory mucosa, which, in addition to the olfactory sensory neurons, contains other cell types such as the supporting cells and progenitor cells. Also, since each OR gene is typically expressed in a very low percentage of the olfactory sensory neurons (∼0.1% in average), this *Olfr17* DNA methylation pattern likely corresponds to that shown by the inactive gene.

**FIGURE 4:**
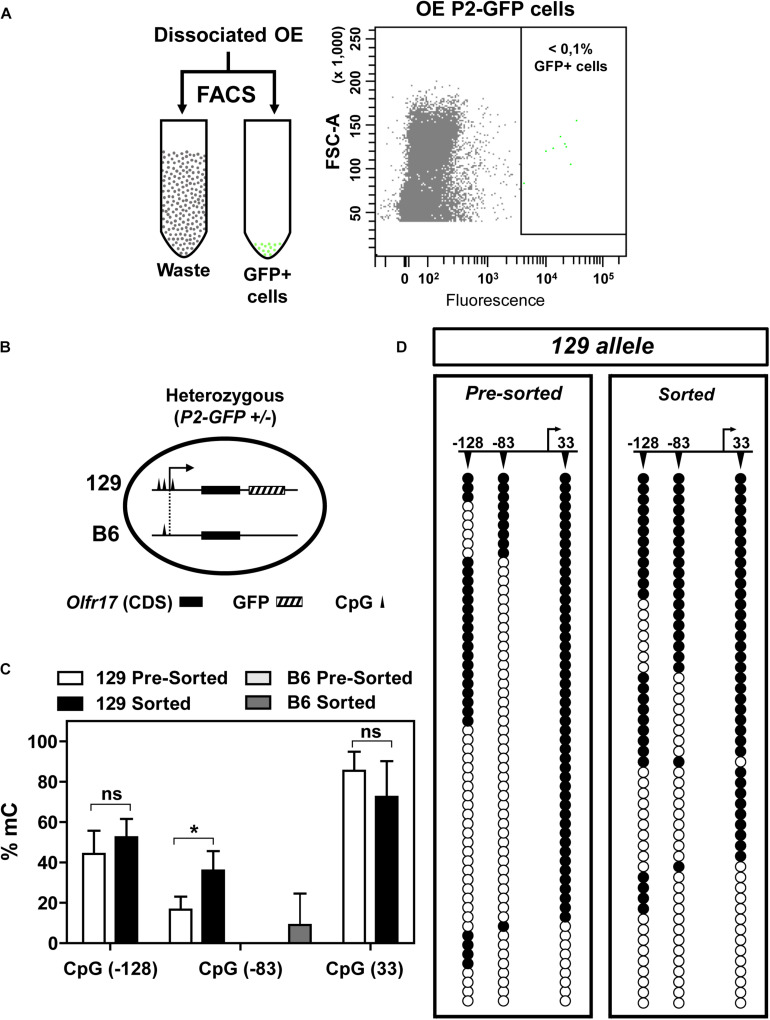
DNA methylation in the 5′ region of *Olfr17* in GFP positive neurons. **(A)** Olfactory mucosa cells were dissociated from P2-GFP mice and submitted to fluorescence activated cell sorting (FACS) in order to isolate GFP+ neurons. The flow cytometry dot plot on the right was obtained when olfactory neurons from the P2-GFP mice were sorted, and the green dots within the square represent GFP+ neurons. **(B)** Schematic representation of the olfactory neurons sorted by FACS, showing that the cells from heterozygous knock-in mice contain one transgenic allele (129 *Olfr17* allele) and one B6 *Olfr17* allele. The vertical lines indicate the positions of the CpGs (–128, –83, and +33) in the promoter region and exon1. The 129 allele contains the three CpGs and the B6 allele contains only CpG –83. **(C)** Comparison of the *Olfr17* methylation frequencies in pre-sorted and sorted neurons in both alleles. In the pre-sorted cells, CpG-83 in the B6 allele showed 0% methylation frequency. Student’s *t*-test, *n* = 3, *P < 0.05. **(D)** Distribution of the methylated CpGs in each sequenced clone from the 129 allele shown in the graph in **(C)**. Open and closed circles indicate unmethylated and methylated CpGs, respectively.

To analyze DNA methylation in the active *Olfr17*, we first separated GFP positive olfactory sensory neurons from heterozygous (P2-GFP^+/–^) knock-in mice by fluorescence-activated cell sorting (FACS) (Figure 4A). Since GFP is co-expressed only with the 129 *Olfr17* allele, these neurons must contain the active 129 *Olfr17* allele, which can be discriminated from the inactive B6 *Olfr17* allele (Figure 4B). Genomic DNA from the pre-sorted and sorted neurons was submitted to DNA bis-seq and the methylation profiles of the 129 *Olfr17* allele promoter region was analyzed. The SNP +34 was used to discriminate between the (GFP containing) 129 *Olfr17* allele and the B6 *Olfr17* allele during DNA bis-seq (Figures 2B, 4B). We found that in the sorted neurons, the CpGs in the 129 *Olfr17* alleles show similar levels of methylation, except for CpG −83, which showed increased level of methylation when compared to pre-sorted cells (Figure 4C). We also found that CpGs in the coding region remain highly methylated, with CpGs showing 80–95% methylation frequencies (data not shown). The CpGs in the 129 *Olfr17* alleles from sorted cells show a random pattern of methylation, with no CpG site being preferentially methylated or unmethylated in the majority of the clones (Figure 4D). Thus, DNA methylation in the promoter region of *Olfr17* is not reduced in GFP positive neurons. Instead, a significant increase in the methylation frequency is observed in one of the three CpGs (CpG −83).

As shown in Figure 2, the B6 allele has only one CpG in the promoter region (CpG −83). One could expect that in the sorted cells, which contains only cells that express *Olfr17* from the 129 allele, the methylation frequency in the B6 allele would be higher than in the pre-sorted cells, which encompass cells expressing *Olfr17* from both the B6 and 129 alleles. Indeed, the B6 allele showed higher CpG −83 methylation levels in the sorted cells than in the pre-sorted cells, however, these methylation levels were very low (0% methylation frequency in pre-sorted cells versus 9% in sorted cells) (Figure 4C).

We also examined cytosine methylation in *Olfr1507* (also known as MOR28), an OR gene that is highly expressed in the B6 olfactory epithelium (11.101 fpm, as determined by Ibarra-Soria et al., 2017), and contains four CpGs in its promoter region. We found that, despite the fact that this is one of most abundantly expressed OR genes, the CpGs in the *Olfr1507* show high methylation frequencies (Supplementary Figure 3). Therefore, high methylation frequencies do not necessarily result in low expression levels. However, analysis of a larger number OR genes would be required to clarify this issue.

### Expression of Neighboring OR Genes in the P2-GFP Mice

**FIGURE 5:**
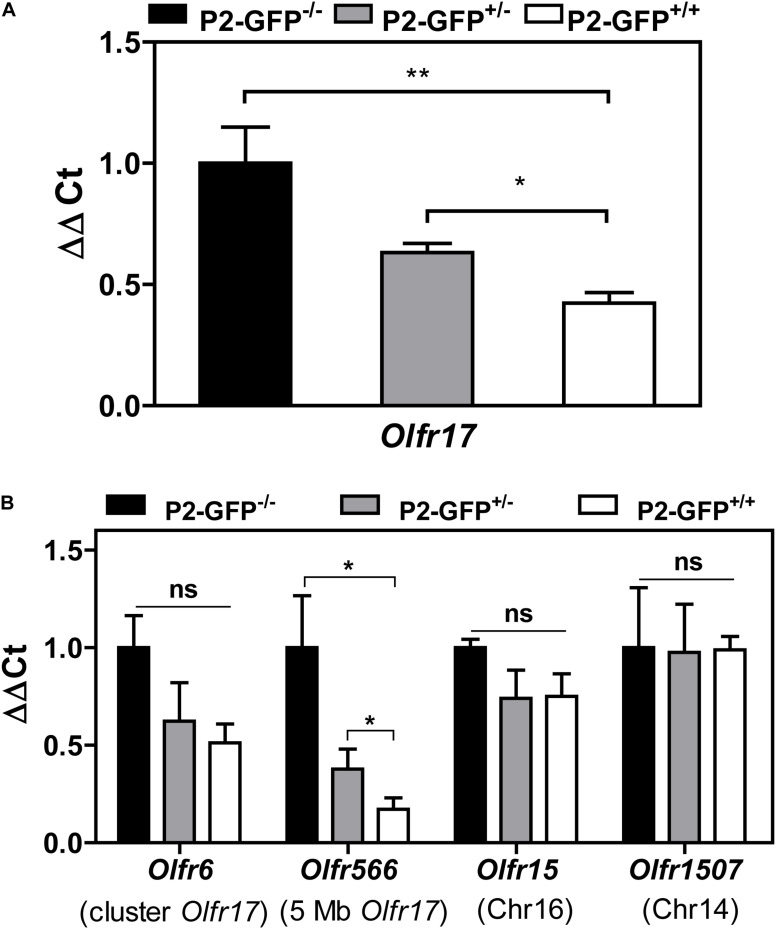
Expression of OR genes located close to *Olfr17* or in other chromosomes in the P2-GFP mice. **(A)** RT-qPCR was performed to determine the relative *Olfr17* gene transcript levels in the olfactory epithelium from P2-GFP^− /−^; P2-GFP^+/−^ and P2-GFP^+/+^ mice. The levels were normalized to β-actin and are shown relative to the expression in homozygous olfactory epithelium. Graph shows mean +/− SEM (P2-GFP^+/+^, *n* = 5; P2-GFP^+/−^, *n* = 4; P2-GFP^− /−^, *n* = 5). *P < 0.05; **P < 0.01. One-way ANOVA followed by Newman–Keuls *post hoc* test or student’s *t*-test. **(B)** RT-qPCR and the same cDNAs as in **(A)** were used to determine the relative transcript levels of *Olfr6*, *Olfr566*, *Olfr15*, and *Olfr1507*. Graph shows mean +/− SEM [P2-GFP^+/+^, *n* = 3 (*n* = 4 for *Olfr6* and *Olfr15*); P2-GFP^+/−^, *n* = 4 (*n* = 3 for *Olfr1507*); P2-GFP^− /−^, *n* = 3]. Not significant: ns, *P < 0.05. One-way ANOVA followed by Newman–Keuls *post hoc* test.

As shown above the B6 and 129 *Olfr17* alleles contain *cis* proximal regulatory regions which differ in sequence and methylation levels. Our RT-qPCR experiments show that in the knock-in siblings, where both *Olfr17* alleles are expressed from the same mixed genetic background (129/B6), the 129 *Olfr17* allele is expressed at lower levels than the B6 *Olfr17* allele. As shown above, the *Olfr17* transcript levels in the olfactory epithelium from homozygotes (P2-GFP^+/+^), where *Olfr17* is expressed from 129 alleles, is significantly reduced when compared to the levels from wild types (P2-GFP^–/–^), where the *Olfr17* is expressed from the B6 alleles (Figure 1B). Accordingly, heterozygotes (P2-GFP^+/–^), where *Olfr17* is expressed from one B6 and one 129 allele, show intermediate levels of expression (Figure 5A).

The variations in the 5′ region of the two *Olfr17* alleles could account for the reduced expression levels of the 129 allele when compared to the B6 allele in the P2-GFP mice. Nevertheless, other factors could also be involved. Selection and stabilization of the expression of an OR gene type was shown to depend on the level of expression of the OR gene, so that ORs that are transcribed at higher levels are able to more efficiently suppress the expression of other OR genes than ORs that are expressed at lower levels (Abdus-Saboor et al., 2016). Therefore, it is possible that the bicistronic P2-IRES-tauGFP mRNA produced from the tagged 129 allele used in our experiments may be less stable and/or less efficiently translated than the untagged 129 *Olfr17* mRNA, leading to reduced levels of *Olfr17* mRNA and *Olfr17* protein in the cells expressing the knock-in allele (Bressel et al., 2016).

We next analyzed the transcript levels of additional OR genes in the same P2-GFP siblings. The experiments were performed with two OR genes located in *cis* to *Olfr17* (*Olfr6*, located in the *Olfr17* cluster and *Olfr566*, located 5 Mb away from *Olfr17*) and two OR genes located in other chromosomes (*Olfr15*, located in chromosome 16 and *Olfr1507*, located in chromosome 14). We found that like *Olfr17*, the OR genes that are in *cis* to *Olfr17* show reduced transcript levels in the heterozygote and homozygote mice (Figure 5B). On the other hand, the OR genes that are located in different chromosomes show similar levels of expression in all three knock-in genotypes (Figure 5B). Therefore, not only the 129 *Olfr17* targeted allele, but other OR genes that are located in *cis* to the targeted allele also show reduced levels of expression.

## Discussion

### DNA Methylation Pattern of *Olfr17*

Technologies that allow for genome wide analysis of DNA methylation in mammals have revealed that the genome is globally methylated at CpG sites, except for the ones which are localized within CpG islands and other regulatory gene regions, which would be bound by proteins involved in transcription and protected from methylation by DNA methyltransferases (DNMTs) (Suzuki and Bird, 2008; Laird, 2010; Jones, 2012). Consistent with these observations, we found that the coding region of the *Olfr17* gene shows high levels of CpG methylation. OR gene promoter regions are usually AT rich sequences and their CpG dinucleotides content is lower than the one observed across the mouse genome (around 1 versus 42%) (Jabbari and Bernardi, 2004; Clowney et al., 2011). Consequently, no CpG islands are present in the promoter regions of the OR genes, but only a small number of CpGs (Clowney et al., 2011). Whether methylation of one or a few CpGs in the regulatory region can affect transcription, remains unclear (Lioznova et al., 2019).

The *Olfr17* gene DNA methylation was similar in the olfactory mucosa and liver (where OR genes are not expressed), indicating that the DNA methylation pattern of this OR gene is not tissue specific. Accordingly, a previous study showed that the CpGs in the coding region of *Ofr151* are also highly methylated, both in the olfactory mucosa and in sperm (Dias and Ressler, 2014). In addition, we found no evident differences in methylation between different developmental stages (embryonic and adult olfactory mucosa).

The role played by DNA methylation at non-CpG sites is not well understood. Genome wide non-CpG methylation was observed in the adult mouse brain (Xie et al., 2012; Guo et al., 2014). The most common type of non-CpG methylation is methylation at CpA sites. Our results show that CpA methylation in *Olfr17* occurs at low frequencies and is randomly distributed throughout the sequence, with no preferentially CpA methylated sites being observed.

### Polymorphisms in the 5′ Region of *Olfr17*

We show that due to sequence variation, the 5′ region of the 129 *Olfr17* allele shows an overall higher level of DNA methylation than the one from the B6 *Olfr17* allele. In general, CpG methylation is thought to repress transcription by directly preventing the binding of transcription factors, or by recruiting histone deacetylases which promote chromatin condensation. However, we found that DNA methylation levels in the 5′ region of the 129 *Olfr17* in olfactory neurons that express *Olfr17*, are similar to the ones obtained from whole olfactory mucosa. Therefore, within the same genomic context (in the transgenic mixed B6/129 background), we observe no correlation between reduced levels of DNA methylation and *Olfr17* expression.

Even though we do not observe a difference in methylation between the active and inactive states of *Olfr17*, we cannot exclude the possibility that DNA methylation levels in regulatory regions may interfere with the probability by which an OR gene is selected for expression. A high-throughput analysis using human genome data from the 1000 Genomes Project revealed a high level of SNPs in the promoter regions of human OR genes (Ignatieva et al., 2014). Whether this high level of polymorphism contributes to differential expression of the OR types across human individuals, is an intriguing question.

### Differential Expression of the 129 and B6 *Olfr17* Alleles

By comparing the expression of the B6 and 129 *Olfr17* alleles in the P2-GFP animals we found that the 129 allele is expressed at lower levels and in a smaller number of olfactory neurons than the B6 allele. These results cannot be attributed to different genomic backgrounds, since the alleles were analyzed on the same mixed B6/129 background. Different possibilities, which are not exclusive, could account for reduced expression of the 129 allele.

In the knock-in mice, the 129 *Olfr17* allele is tagged, while the B6 one is not. To what extent expression of a bicistronic mRNA affects OR gene expression, is not clear yet. One way to address this point would be to compare the expression of wild type and tagged OR alleles in the same neurons. In a previous work, expression of a wild type 129 *Olfr1507* allele and a tagged 129 *Olfr1507* allele in the olfactory epithelium from 129 MOR28-IRES-gap-GFP/129 MOR28 mice was compared. In these experiments, a slight reduction in the probability of choice was observed for the tagged allele, when compared to the wild type allele [51.1% of neurons expressed *Olfr1507* and 47.2% of the neurons expressed both *Olfr1507* and GFP (Low and Mombaerts, 2017)]. Therefore, in this case, even though the presence of the tag contributed to a reduction in the probability of OR gene choice, this effect was very small, when compared to the ones observed in our experiments: a 64% reduction in the *Olfr17* transcript levels (as measured by RT-qPCR) and a 30% reduction in the number of *Olfr17* expressing neurons in P2-GFP^+/+^ mice when compared to P2-GFP^–/–^.

The observations above suggest that the burden of expressing the GFP tag may not be the only reason for the reduced *Olfr17* transcript levels we observe in the knock-in mice. The results could also be explained by a competition process between the alleles, where transcription of the B6 allele would be more permissive than the transcription of the 129 allele, due to sequence/epigenetic variations in the 5′ regions of these alleles. A similar type of competition has already been observed in heterozygous cells containing both the B6 and 129 *Olfr1507* alleles. In this case, there was also a preference for the expression of the B6 allele (Low and Mombaerts, 2017). Alternatively, higher expression levels from the B6 allele in the heterozygous cells, could suppress expression of the 129 allele, through a post selection refinement (PSR) process (Abdus-Saboor et al., 2016).

We found that reduced expression is not limited to *Olfr17*, but additional OR genes that are located in *cis* to the *Olfr17* locus are also expressed at lower levels in the knock-in mice. These results suggest that *cis* effects of the integrated DNA interfere with the expression of the neighboring genes. Altered chromatin configuration and other *cis-*regulatory interactions could contribute to these effects (Olson et al., 1996). Notably, based on the work by Ibarra-Soria et al., 2017, the expression levels of the nearby *Olfr6* and *Olfr566* are lower in 129 wild type mice than in B6 wild type mice. It is not clear yet how much of the flanking regions from the targeted locus in the P2-GFP mice are still derived from the 129 background. These regions could include not only OR genes, but also enhancers and other regulatory sequences. Therefore, a comparative analysis of the whole *Olfr17* locus and its flanking regions in the wild type and knock-in mice should contribute to unveil the regulatory mechanisms responsible for the differences in *Olfr17* expression. Finally, these findings highlight the importance of considering that such *cis*-acting effects may occur when using gene-targeted animal models in the study of OR genes.

### *Olfr17* Expression in the Wild Type Strains

Our study raises important questions regarding differential OR gene expression between diverse wild type strains. Why is *Olfr17* expressed at higher levels in the 129-mouse strain compared to the B6 strain? Does the 129 *Olfr17* promoter region in wild type 129 mice show lower frequencies of methylation than in P2-GFP mice, as it would be expected? Our results with *Olfr1507* argue against a simple and linear correlation between promoter methylation status and expression levels of OR genes. Olfactory neurons have a characteristic form of nuclear organization, and both in *cis* and inter-chromosomal interactions are required for the correct expression of the OR genes (Clowney et al., 2012; Armelin-Correa et al., 2014; Monahan et al., 2019). DNA methylation and/or other epigenetic factors could result in differential 3D chromatin organization in the olfactory nuclei from the different mouse strains, and produce distinct *cis* and *trans* effects that could lead to differential *Olfr17* expression levels. The understanding of how genetic variations among mouse strains contribute to differential OR gene expression, should reveal important aspects of OR gene regulation.

## Data Availability Statement

The original contributions presented in the study are included in the article/Supplementary Material, further inquiries can be directed to the corresponding author.

## Ethics Statement

The animal study was reviewed and approved by the University of São Paulo Chemistry Institute’s Animal Care and Use Committee, under the protocols number 19/2013 and 60/2017.

## Author Contributions

AL, MN, TN, and BM designed the experiments and analyzed the data. AL, MN, and TN performed the experiments. AL and BM wrote the manuscript. All authors contributed original ideas to the research and agreed on the final version of the manuscript.

## Conflict of Interest

The authors declare that the research was conducted in the absence of any commercial or financial relationships that could be construed as a potential conflict of interest.
