# Supplementary material for: Genetic Background Effects on the Expression of an Odorant Receptor Gene
Source: Front Cell Neurosci. 2021 Feb 25;15:646413. doi: 10.3389/fncel.2021.646413 (PMC7947310; doi:10.3389/fncel.2021.646413)
Supplement: Supplementary file 1 [file Data_Sheet_1.PDF]

Supplementary Material

1 Supplementary Figures

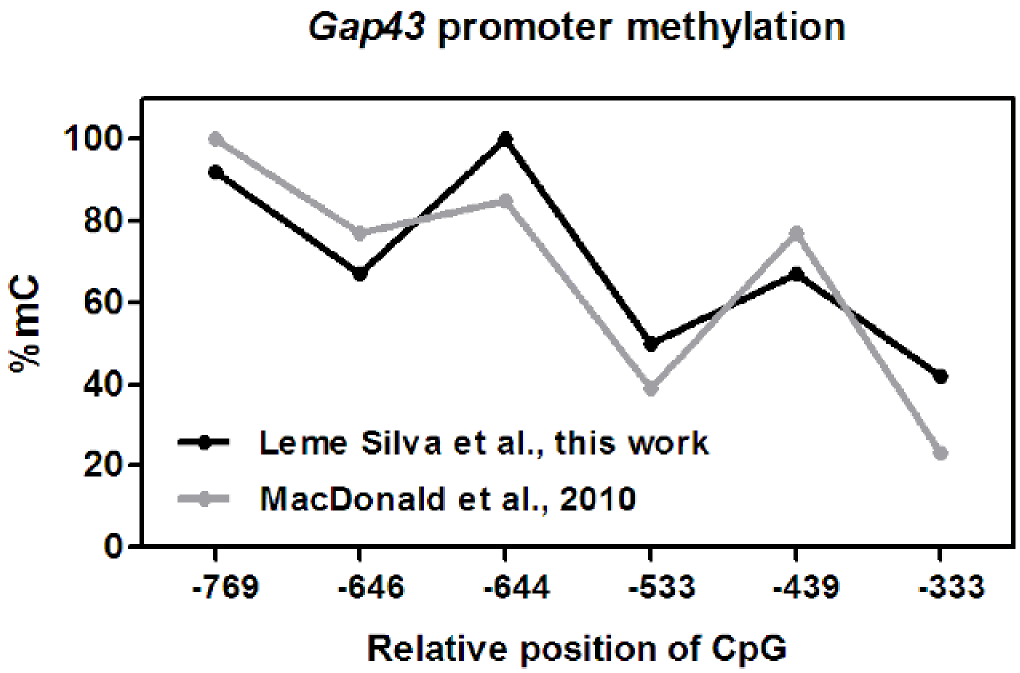

Supplementary Figure 1. The DNA bisulfite sequencing method was validated using the DNA methylation profile of the Gap43 promoter as a positive control. The results are compared to the one previously reported (Macdonald et al., 2010).

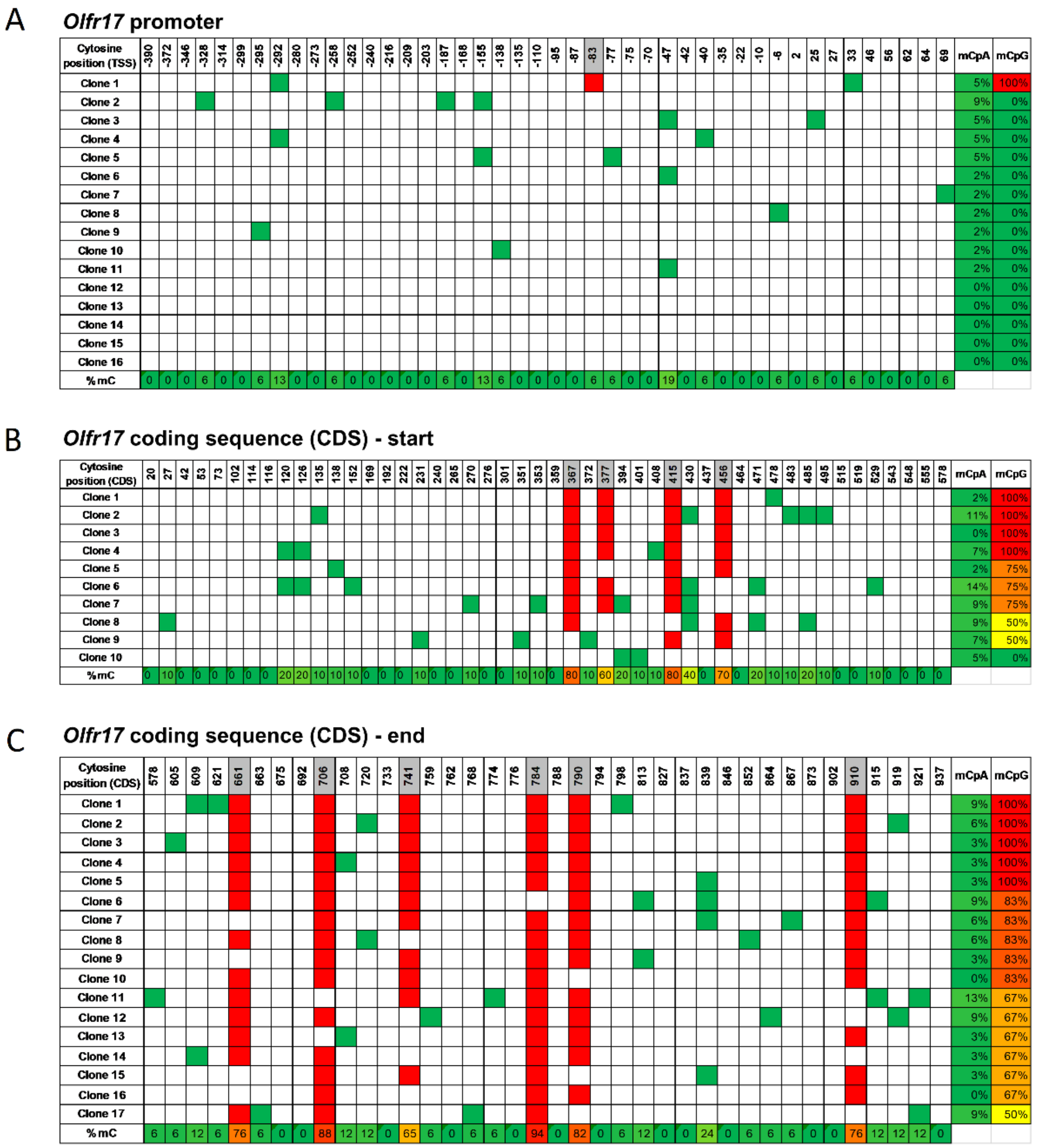

Supplementary Figure 2. Representative cytosine methylation results obtained for the Olfr17 promoter region (A) and for the first half (B) and second half (C) of the coding region are shown. The positions of the cytosines relative to the TSS or in the coding region are shown in the first line of the table, where gray boxes represent cytosines in the CpG dinucleotide context, and the empty boxes represent cytosines in the CpA dinucleotide context. Filled squares in the table indicate a methylation on a cytosine, the green squares represent methylated CpAs and the red squares represent methylated CpGs. The methylation frequency (%mC) corresponds to the number of clones that retain cytosine after bisulfite conversion in each position divided by the total number of clones analyzed.

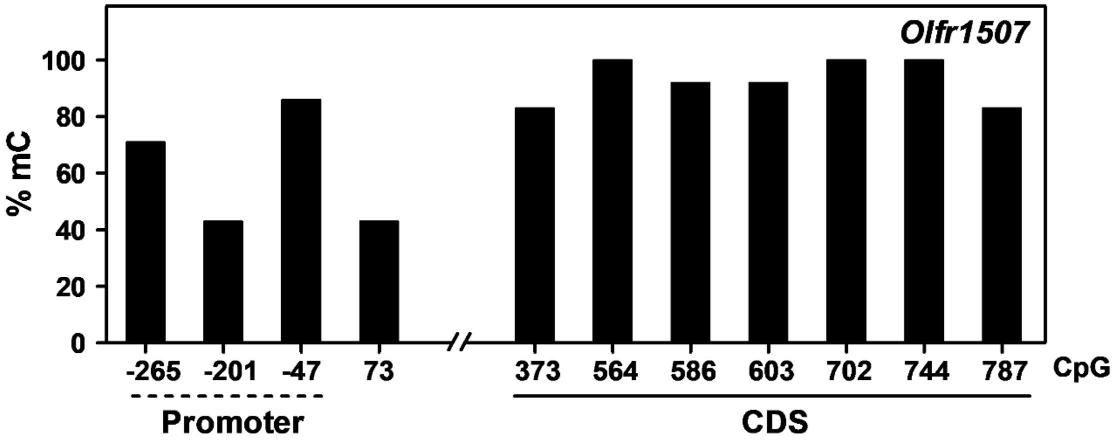

Supplementary Figure 3. CpG methylation in the promoter and coding regions of *Olfr1507*.
